# Effects of whole body vibration exercise on lumbar-abdominal muscles activation for patients with chronic low back pain

**DOI:** 10.1186/s13102-020-00226-4

**Published:** 2020-12-10

**Authors:** Yulin Dong, Huifang Wang, Yan Zhu, Binglin Chen, Yili Zheng, Xiaochen Liu, Jun Qiao, Xueqiang Wang

**Affiliations:** 1 Department of Treatment, The Second Rehabilitation Hospital of Shanghai, 25 Changjiang RD, Shanghai, China; 2 Yang Zhi Affiliated Rehabilition Hospital of Tongji, Shanghai, China; 3 The Second School of Clinical Medicine, Xuzhou Medical University, Xuzhou, Jiangsu China; 4 Department of Sport Rehabilitation, Shanghai University of Sport, 399 Changhai RD, Shanghai, China

**Keywords:** Vibration, Low back pain, Abdominal muscles

## Abstract

**Background:**

Whole body vibration (WBV) training as an intervention method can cure chronic low back pain (CLBP). Different WBV parameters exert different effects on lumbar-abdominal muscle performance. Currently, there is a lack of study researched the influence of WBV training on patients with CLBP by lumbar–abdominal muscle activity. Therefore, this study aimed to investigate how WBV and exercise and their interactions influence lumbar-abdominal muscle activity in patients with CLBP.

**Methods:**

a group of ambulatory patients with chronic low back pain. Muscle activities of the multifidus (MF), erector spinae (ES), abdominal oblique externus muscle (AOE) and the rectus abdominis muscle (RA) were measured by surface electromyography, whereas participants performed 4 different exercises (single bridge, plank, side stay and V crunch) during three whole body vibration conditions and a no-vibration condition in a single experimental session.

**Results:**

Compared with the same exercises without whole body vibration, muscle activity increased when whole body vibration was added to the exercises. MF; the WBV frequency (*P* = 0.002,) and exercise (*P* < 0.001) presented significant effects on the root mean square of MF, whereas exercise * frequency (*P* = 0.044) also resulted in significant interaction effects. ES: the significant differences were detected at WBV frequency (*P* < 0.001), exercise (*P* < 0.001), the interaction effect of exercise and frequency (*P* = 0.225) was no significant. RA: the significant difference was detected at WBV frequency (*P* = 0.018), the effect of exercise (*P* = 0.590) and the exercise * frequency interaction (*P* = 0.572) were no significant. AOE: the significant difference was detected at WBV frequency (*P* < 0.001), the effect of exercise (*P* = 0.152) and the exercise * frequency interaction (*P* = 0.380) were no significant.

**Conclusion:**

Adding whole body vibration to exercise could increase muscle activation of lumbar–abdominal muscle in patients with CLBP. The optimum frequency for lumbar–abdominal muscles is 15 Hz. The best exercises include plank for multifidus and erector spinae, V crunch for rectus abdominis and single bridge for abdominal oblique externus.

**Clinical registration:**

Trial registration: ChiCTR-TRC-13003708. Registered 19 October 2013.

**The code of ethical approval:**

2014008.

## Background

Whole body vibration (WBV) has been common among scientific studies in the last decade. WBV has emerged as a new type of neuromuscular training method that can effect muscle strength and power [1], electromyographic activity [2], neuromuscular reflex [3], and postural control [4].

WBV training has a wide range of vibration parameters, including vibration types (alternating and vertical vibrations), frequency, amplitude, time and body posture. Several clinical experiments have investigated the influence of different WBVs on lower limb muscle activation; the results indicated that different exercises induced varying degrees of leg muscle activity [2, 5, 6]. Low-intensity WBV training is more suitable for muscle activation than high-intensity WBV [2]. Muscle activation also depends on the interaction between frequency and body position [7]. According to these studies, different parameters exert different effects on muscle performance.

As for lumbar–abdominal muscles, combining routine lumbar and abdominal exercises, WBV training in healthy adults showed that static trunk muscle exercises could induce a low-to-moderate muscle activation [8]. Different parameters of WBV training could affect lumbar-abdominal muscle activities in healthy adults [9, 10]. Richard, et al., reported that they found significantly more average and peak-to-peak estimated torque at almost all frequencies for vibration vs staticiitting [11].High vibration frequencies can lead to enhanced exercise benefits within an appropriate frequency range for lumbar-abdominal muscle in healthy adults [10]. Muscle activity during Stochastic resonance whole body vibrations (SR-WBV) is reasonably low and comparable to core strength stability exercises, sensorimotor training and “abdominal hollowing” in water [12]. In addition, flexor muscle weakening and imbalance of muscle strength are important cause of chronic low back pain (CLBP) [13, 14]. Strengthening lumbar muscles is an important objective of CLBP treatment [15]. Muscle activation is one of the main indicators to evaluate muscle performance [10]. Therefore, lumbar–abdominal muscle activation is also closely related to chronic low back pain (CLBP). Several clinical intervention studies reported that WBV training as an intervention method can reduce CLBP effectively and improve quality of life [16–18]. To our knowledge, no study researched the influence of WBV training on patients with CLBP by lumbar–abdominal muscle activity. Furthermore, no standard clinical guidelines are available for WBV training in relation to curing CLBP.

Several clinical experiments have reported the effect of WBV on muscle activation [2, 19], and most of the results indicated that surface electromyography (sEMG) amplitude increases by adding vibration to multiple exercises [20]. sEMG of lumbar–abdominal muscle represents a kind of bioelectrical signal, that is the sum of applicable signal in time and space. To a certain extent, sEMG can reflect neuromuscular activity. One of the sEMG indicators is root mean square (RMS) amplitude, which is the instantaneous RMS amplitude of EMG over a period of time [21]. RMS can be used to evaluate the level of lumbar-abdominal muscle activity under different movements, loads and frequencies [22, 23].

To solve these knowledge gaps, this study observed how different WBV conditions affected the lumbar–abdominal muscle activation in patients with CLBP. The study hypothesised the following: (1) WBV training with exercises can increase muscle activation compared with the same exercise without WBV; (2) different frequencies cause varying effects on muscles, with higher frequencies inducing higher neuromuscular activity than lower WBV frequencies; (3) different exercises exert various effects on specific lumbar–abdominal muscles; (4) a significant interaction occurs between exercise and vibration frequency in lumbar–abdominal muscle activation.

## Methods

### Participants

This study designed an clinical research with repeated measures [2, 24]. Twenty-one participants were recruited from the Shangti orthopaedic hospital and the second rehabilitation hospital in Shanghai. All the subjects were 20 years old or over and had CLBP. The inclusion criteria were:(1) Ages from18 to 60 years old, (2) persistent pain for 3 months at least or intermittent pain for 3 times a week over a period of 3 months, (3) there were no anaesthesia and abnormal lower limb reflexes. Exclusion criteria were: (1) undergone previous surgery, dislocation, fracture, rheumatoid arthritis, ankylosing spondylitis and disc pathology (2) organic diseases, metabolic diseases, cardiovascular diseases, progressive neurological deficits or sever osteoporosis, (3) pregnant or lactating, (4) sever hypertension, (5) Numerical Pain Scale (NPS, from 1 to 10) over 8, (6) did WBV exercise in last 3 months, (7) other diseases, advisable to participate this study based on the judgment of patients or physicians, (8) individuals who had any of the following situations will be excluded.

Prior to inclusion, we asked for all the participants to inform consent. Before attending the study, all participants would do a questionnaire about the following details: basic information, NPS, oswestry disability index (ODI) and daily exercise habits.

### Experimental protocol

The study was approved by the Ethics Committee of Shanghai University of Sport. We carried out all the experimental programs in the same laboratory and collect complete data of each subjects in one single experiment session.

**Fig. 1:**
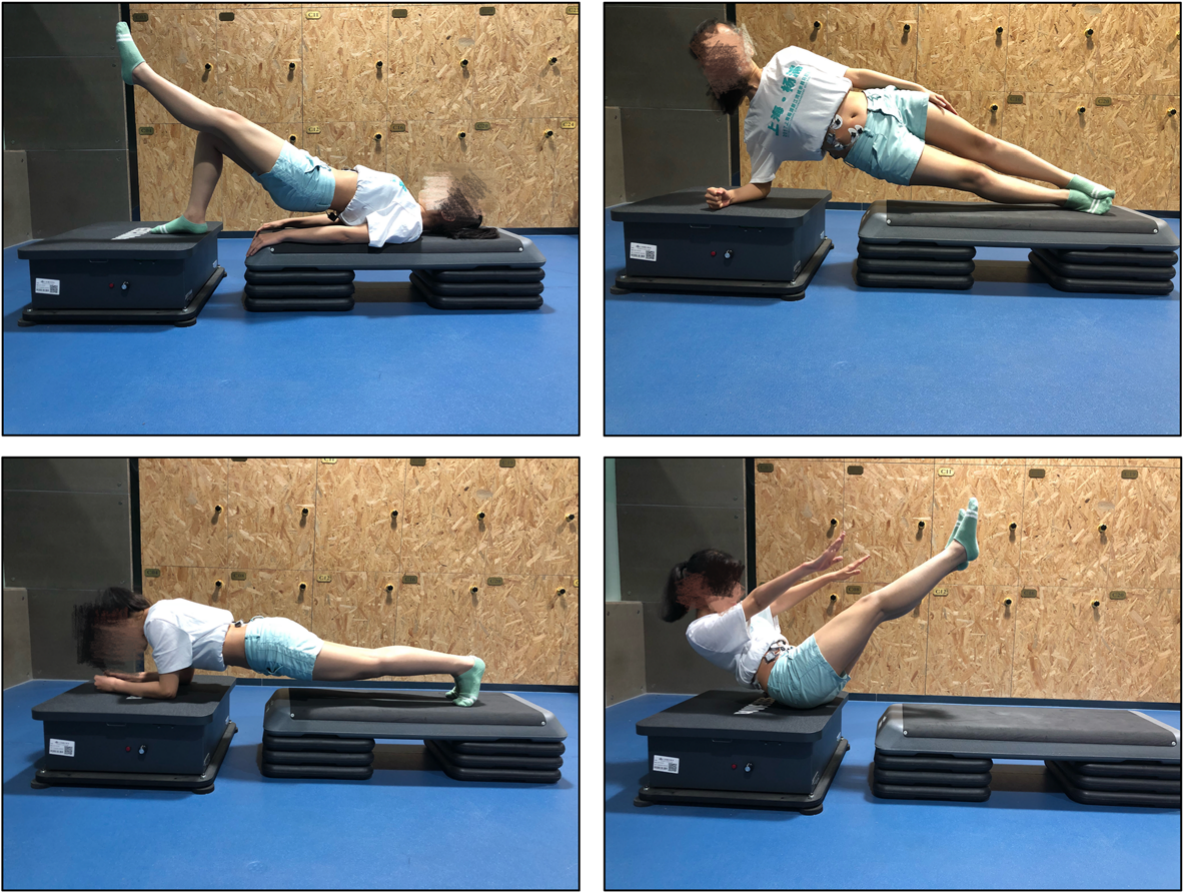
Four exercises on the platform: single bridge, plank, side stay and V crunch

A WBV machine generating vertical vibrations was used (AV-009; BODYGREEN, Taiwan, China) at a vibration frequency range of 5-35 Hz and amplitude of 2 mm. The three frequencies (5 Hz, 10 Hz and 15 Hz) and one amplitude (2 mm) were selected in this study. Also, a control condition without WBV was also tested. All participants were demanded to practice 4 kinds of exercises, producing 16 testing conditions in all. These exercises were as follows (Fig. 1). For single bridge, the participants lay on the platform, with one leg bent over the vibration platform and the other leg lifted straight off the platform. The patients extended their hip joint and back off the platform. For planking, both elbows were placed on the vibration platform, whereas the back, hip and both legs stay straight off the platform. For the side stay, the participants lay on their side on the platform, with a single elbow placed on the vibration platform. For V crunching, the patients rolled up their abdomen, pushed their shoulders towards the pelvis, and situated their upper hip on the vibration platform. The participants were randomly subjected to 16 testing conditions. Before data collection, all the participants must be familiarized with all the target exercises. Participants were instructed to perform each exercise under three vibration frequencies and no vibration. In total, each exercise was performed for 130 s (each exercise condition lasted for 10 s, with a 30 s break between two frequencies); the patients rested for 5 min between two exercises. During the exercises, the experimenter recorded the lumbar–abdominal muscle sEMG signals of the participants. Each participant practiced the sequence of the vibration frequency and exercises randomly.

EMG data were collected by MyoResearch XP Master Software Version 1.07.17(Noraxon Inc., Scottsdale, USA) with sampling frequency of 1500 Hz. After we use medical alcohol and razor to reduce skin impedance. The electrodes (Silver chloride, Shangqiankang medical equipment co. LTD) were put in place referring to the updated introduction for sEMG [25].We choose the lumbar and abdominal muscles on the side of severe pain were multifidus (MF), erector spinae (ES), rectus abdominis (RA) and abdominis oblique externus (AOE) respectively. The placement of the electrode is as follows: (1) MF: 2–3 cm from the midline at the level of L5, putted on and aligned with a line from caudal tip posterior spina iliaca superior to the interspace between L1 and L2; (2) ES: An electrode was placed 2 cm apart from the midpoint of the bilateral iliac ridge connection; (3) RA: 2–3 cm lateral from the midline on the second segment of the muscle; (4) AOE: at half of the line from the anterior spina iliaca superior and the tip of the 11th rib [26].

### Data analysis

All original data were performed by MyoResearch XP Master Software Version 1.07.17 (Noraxon Inc., Scottsdale, USA). First, the original EMG signals were processed with a 10 and 500 Hz noise by utilizing Mat Lab [9]. For the four exercises, the middle 5 s of the data was used to calculate the EMG root mean square (EMGrms), which represents the magnitude of muscle activity. A higher RMS value indicates more muscle activity. For the standardization of sEMG data, the value of RMS under WBV was compared with that under no-WBV, which was considered as the degree of muscle activity during WBV training [2].

### Statistical analysis

Statistical analyses were conducted with SPSS 17.0 and Microsoft Excel 2007. Two-way ANOVA with repeated measures was used to explore the effect of different WBV conditions for each muscle. If the sphericity assumption (Mauchly’s test) was violated, we would use the Greenhouse–Geisser epsilon adjustment. The Shapiro-Wilk test was used to assess the normality of the descriptive data. Statistical analyses were conducted as follows: (1) the main effect of frequency was determined by any conspicuous difference in muscle activation observed amidst the three different frequencies measured; (2) the main effect of exercise was determined by any conspicuous difference in muscle activation observed amidst the 4 different exercises measured; (3) and the frequencies × interaction effect: whether any the interaction effect of WBV frequencies and exercise forms. F-ratio (F) represents the each of main effects, and then it is relative to a critical F-value to evaluate its significance. We determined the muscle activation that was deeply influenced by WBV and used post-hoc analysis (paired t-test) with Bonferroni adjustment for evaluation. The statistical significance level was set at *p*<0.05.

## Results

### Demographics

Twenty-one patients with CLBP attended in the study (11 men, mean age, 22.4 ± 2.62 years). All the patients were recorded demographics by the same physical therapist (Table 1), and all the statistics expresses by mean and standard deviation (SD). Table 2 showed the muscle activities during different exercises with no-WBV/WBV training.

**Table 1:** Demographics of participants (Mean ± SD)

	Mean ± SD
Age, year	22.4 ± 2.62
Gender (Women/Man)	10/11
Weight, kg	61 ± 9.45
Height, cm	168.86 ± 8.3
NPS(0–10)	3 ± 1
ODI (0–100)	20.77 ± 3.73

**Table 2:** Muscle activation measured in the no-vibration/vibration condition (RMS, Mean ± SD)

	Frequency (HZ)	Single bridge (μV)	Plank (μV)	Side stay (μV)	V crunch (μV)
Multifidus muscle	0	67.45 ± 6.15	13.15 ± 4.67	13.64 ± 3.23	23.72 ± 4.06
	5	70.56 ± 5.61	24.53 ± 4.75	22.14 ± 3.16	48.51 ± 6.25
	10	93.45 ± 6.39	32.02 ± 3.22	27.13 ± 4.08	52.16 ± 5.22
	15	84.47 ± 4.28	45.02 ± 5.43	34.37 ± 4.85	56.72 ± 4.01
Erector spinae muscle	0	67.28 ± 5.91	11.65 ± 3.22	13.13 ± 3.59	35.60 ± 9.70
	5	70.87 ± 5.82	17.18 ± 2.18	18.65 ± 2.63	64.73 ± 6.12
	10	74.39 ± 8.52	27.51 ± 3.90	22.66 ± 2.48	71.23 ± 2.23
	15	80.21 ± 3.24	33.65 ± 2.98	25.50 ± 1.68	92.56 ± 5.36
Rectus abdominis muscle	0	11.26 ± 1.86	88.62 ± 7.50	23.02 ± 4.72	89.47 ± 8.03
	5	20.09 ± 2.63	98.23 ± 5.23	28.34 ± 4.10	163.93 ± 7.02
	10	27.43 ± 3.57	135.91 ± 3.60	36.93 ± 3.69	191.24 ± 8.10
	15	35.13 ± 3.73	171.52 ± 4.25	43.96 ± 4.32	231.4 ± 10.37
Obliques externus muscle	0	16.63 ± 2.97	39.27 ± 3.92	21.41 ± 5.68	42.95 ± 4.95
	5	25.58 ± 1.55	56.7 ± 5.15	28.35 ± 4.22	73.98 ± 6.25
	10	41.02 ± 3.30	66.98 ± 2.89	40.15 ± 2.02	112.92 ± 3.65
	15	47.56 ± 4.94	72.12 ± 5.48	49.74 ± 4.03	101.64 ± 10.08

### Muscle activity of Multifidus

**Fig. 2:**
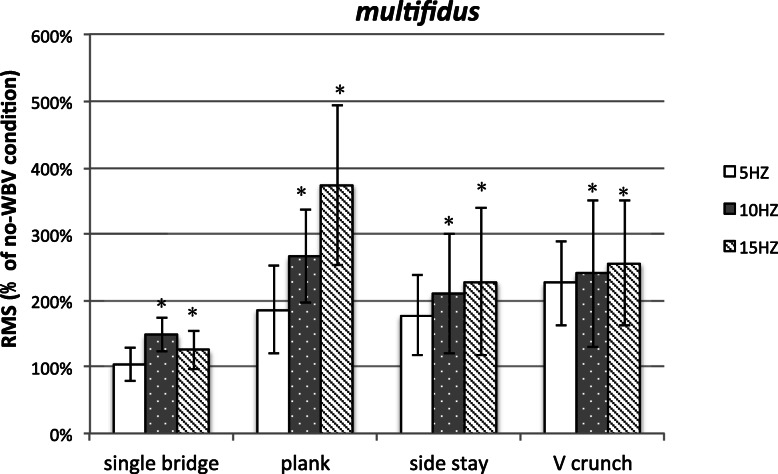
Post-hoc analysis of the muscle activity of MF during different WBV conditions. The degree of muscle activation is described as a percentage (%) of no-WBV conditions during the same exercises. Standardised error of the mean is expressed by error bars. * Significant muscle activation comparison with 5 Hz frequency (paired t-test; *p* ≤ 0.05). Data description is used for all figures

The value of RMS of MF increased compared with that under the same exercise without WBV (total average, 208.7%). The significant differences were detected at WBV frequency (*P* = 0.002,F = 10.736), exercise (*P* < 0.001,F = 10.799) and the exercise * frequency interaction (*P* = 0.044,F = 3.328). It showed that the effect of WBV frequency on the value of RMS of MF was related to exercise. Then, the RMS of MF was highest when the WBV condition was in plank exercise with 15 Hz (Fig. 2).

### Muscle activity of erector Spinae

**Fig. 3:**
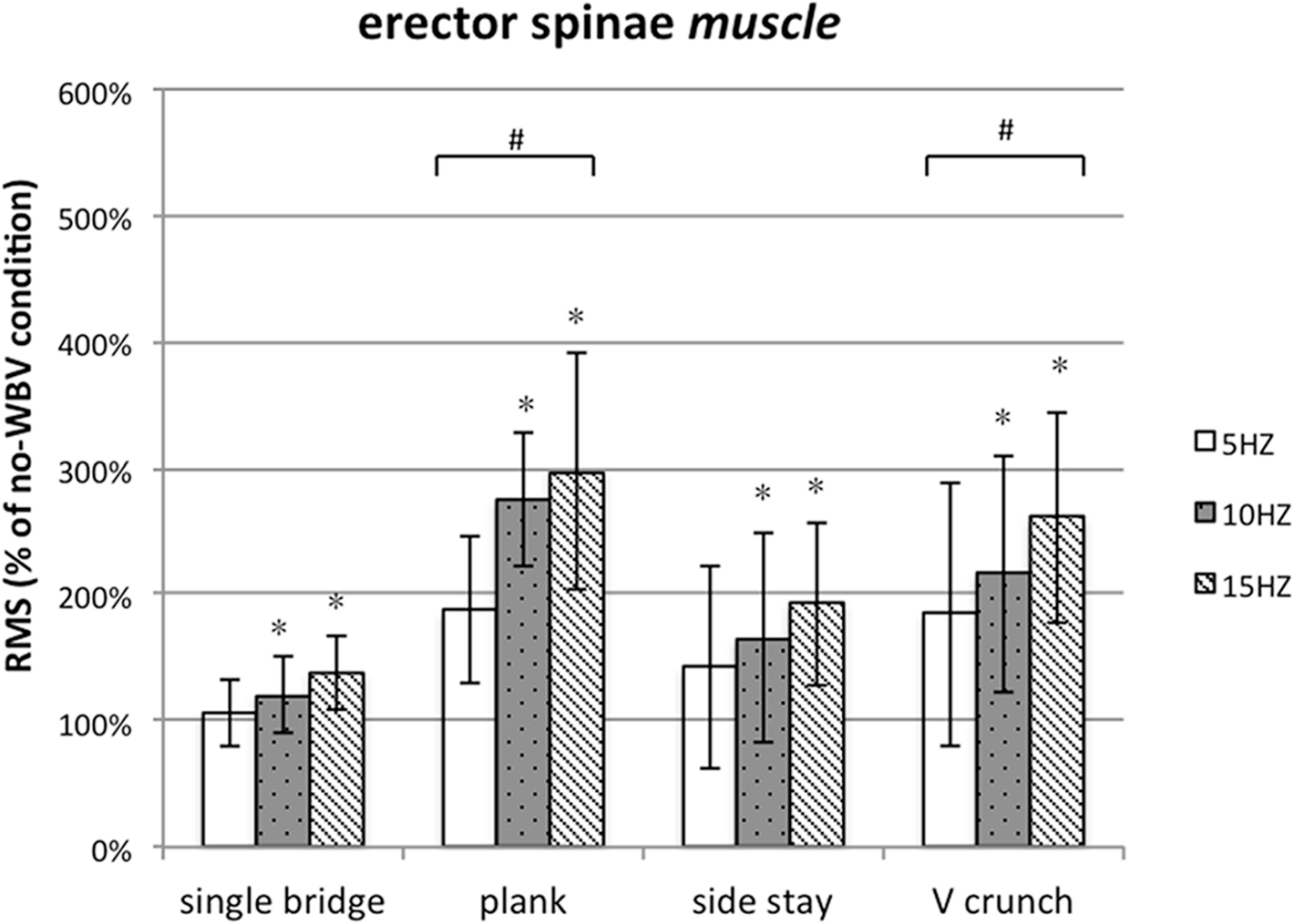
Post-hoc analysis of the muscle activity of ES during different WBV conditions. # Significantly muscle activation comparison with single bridge (paired t-test; *p* ≤ 0.05)

The value of RMS of ES increased compared with that under the same exercises without WBV (The total average, 190%). The significant differences were detected at WBV frequency (*P* < 0.001, F = 12.958), exercise (*P* < 0.001, F = 5.967). The interaction effect of exercise and frequency (*P* = 0.225, F = 1.388) on ES was no significant, indicating that the effect of WBV on ES was not related these factors. Then, using paired comparisons between exercises, the result found that there were significant among single bridge, plank and the V crunch. The highest muscle activation was induced by plank exercise. In the same way, 15 Hz was the best frequency for muscle activation (Fig. 3).

### Muscle activity of rectus Abdominis

**Fig. 4:**
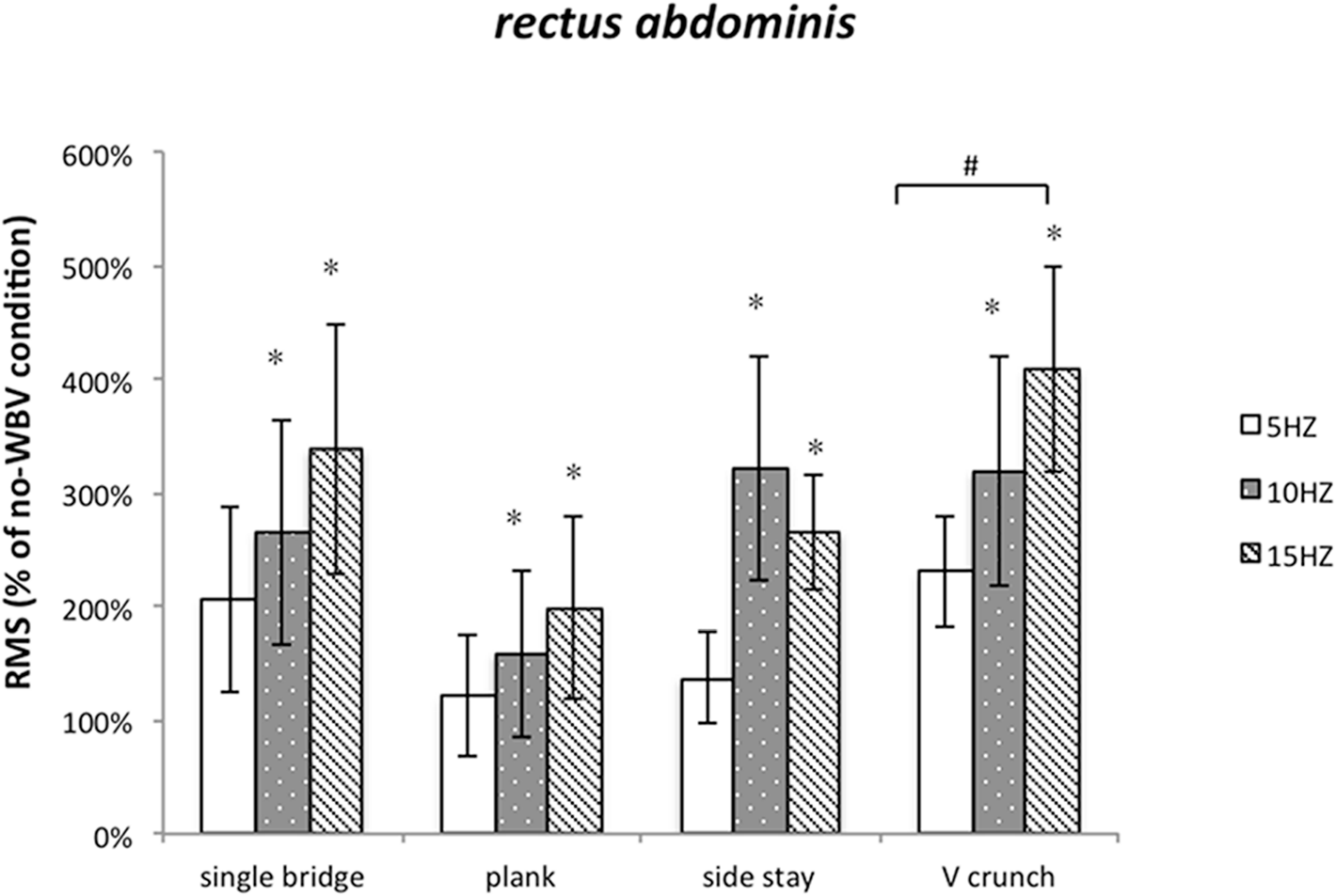
Post-hoc analysis of the muscle activity of RA during different WBV conditions. # Significantly muscle activation comparison with plank (paired t-test; *p* ≤ 0.05)

The value of RMS of RA increased compared with that under the same exercises without WBV (The total average, 247.1%). The significant difference was detected at WBV frequency (F = 6.555, *P* = 0.018). The effect of exercise (F = 0.513, *P* = 0.590) and the exercise * frequency interaction (F = 0.525, *P* = 0.572) were no significant, expressing that effect of WBV frequencies on RA was not relate with exercises. Then, we found 15 Hz can induce highest degree of muscle activation by paired comparisons (Fig. 4). Among the exercises, there was significance between V crunch and plank, V crunch can induce highest degree of muscle activation.

### Muscle activity of abdominal oblique Externus

**Fig. 5:**
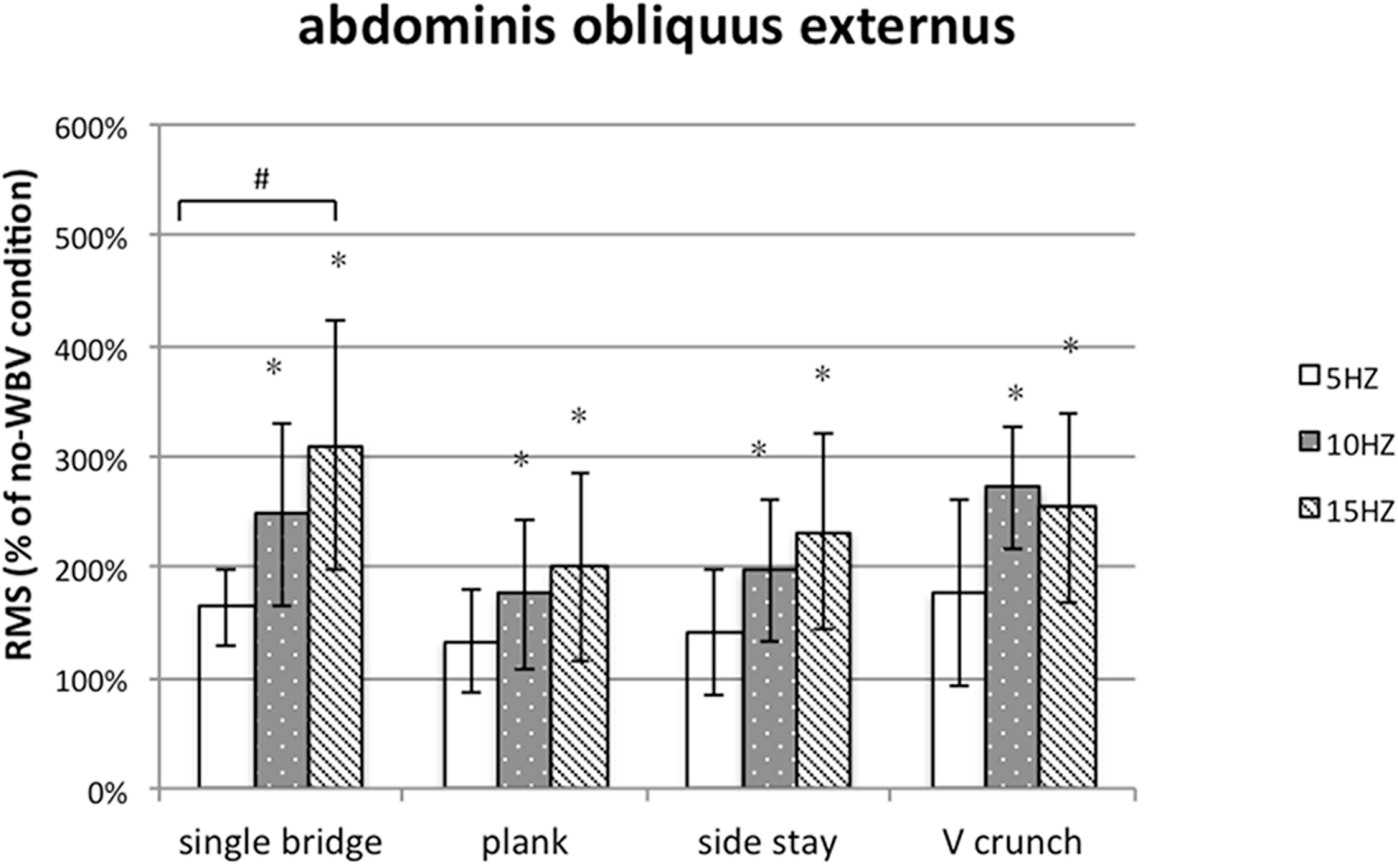
Post-hoc analysis of the muscle activity of AOE during different WBV conditions. # Significantly muscle activation comparison with plank (paired t-test; *p* ≤ 0.05)

The value of RMS of AOE increased compared with that under the same exercises without WBV (The total average, 206.8%). The significant difference was detected at WBV frequency (F = 15.294, *P* < 0.001). The effect of exercise (F = 2.010, *P* = 0.152) and the exercise * frequency interaction (F = 0.975, *P* = 0.380) were no significant, it showed that effect of WBV frequencies on RA was not relate with exercises. Then, we found 15 Hz can induce highest degree of muscle activation by paired comparisons (Fig. 5). Among the exercises, there was significance between single bridge and plank. Single bridge can induce highest degree of muscle activation.

## Discussion

This study aimed to identify the effect of WBV on lumbar abdominal muscle activities in patients with CLBP. Using different combinations of vibration parameters, this study could provide important ideas for clinical treatment or research to attention on the interaction effects of exercise and frequencies.

### Effect of WBV on lumbar-abdominal muscle activation

Results of our study powerfully supported the first hypothesis that adding WBV to exercises could increase muscle activation in MF (the total average, 208.7%), ES (the total average, 190%), AOE (the total average, 206.8%) and RA (the total average, 247.1%). In general, our study showed that muscle activation of patients with CLBP was similar to that of previous studies on healthy adults [9, 26]. For example, the authors evaluated the trunk muscle activation in different static exercises on the vibrating platform, and their results revealed that WBV led to increased muscle activation by 1.6% ± 1.4% maximal voluntary contraction (MVC) in MF and 46.4% ± 14.9% MVC in RA [26]. These data showed that WBV could induce lumbar–abdominal muscle activity. Other factors would affect the degree of muscle activation [9]. For example, healthy individuals performed single bridge exercise with WBV training, resulting in higher degree of ES activation than MF activation [26]. This result differed from the findings of our study, in which CLBP patients conducting single bridge exercise induced the highest AOE activation.

Considering all the evidence gathered from these findings and previous studies, WBV training could be popular among individuals with poor muscle strength, and long-term training would result in more gains [27]. Second, the effect of WBV on muscle activation depends on the body position, types of participants and vibration frequency [2]. Third, we should be rigorous on comparing different populations. The EMG presents individual differences, such as skin interference, body posture and electrode position. Given these uncontrollable factors, we should conduct data standardisation according to certain reference values, such as the value of sEMG in no-WBV condition or MVC. This design uses the value of sEMG in no-WBV condition as reference to ensure reliable conclusions [2].

### Effect of different frequencies on lumbar-abdominal muscle activation

The results of this study revealed that a higher frequency (15 Hz) induces higher neuromuscular activity than lower WBV frequencies (5 and 10 Hz), thus supporting the second hypothesis.

During WBV, the body of an individual is stimulated by appropriate mechanical stimuli. The related mechanism possibly utilises mechanical vibration and external resistance loads to stimulate muscle spindles; this training method improves neuromuscular function by inducing elevated muscle contractile activities and central nervous system adaptation [28]. These study findings were similar to those of several previous studies. For example, Brigitte et al. reported that vibration led to increased RMS value of MF by 26% when compared with that under no vibration [26]. Xueqiang et al. performed a clinical study about the effect of different platforms on lumbar–abdominal muscle activation. They reported that unstable platforms could induce higher activation than stable ones when healthy subjects performed various kinds of exercise [23]. Desai et al. studied the rate of lumbar–abdominal muscle activation (RMS% MVC) in patients with CLBP and healthy people; the results demonstrated that the unstable platform is a good choice for inducing muscle activation [29]. The above evidence all suggest that stimulation of unstable planes or vibration platform increases muscle activity. The practical significance of RMS is as a statistical characteristic of EMG showing muscle activities [21]. When the body is on the vibration platform, the muscle induces more muscle spindles to resist external loads and maintain body balance. To a certain extent, a higher RMS value indicates more muscle activity. Our results showed that high vibration frequency is better for lumbar–abdominal muscle in patients with CLBP than low vibration frequency.

Study have shown the effect of different vibration frequencies on the activation of lumbar and abdominal muscles, revealing no significant differences between different vibration frequencies during bridge exercise [30]. The effect of WBV training (less than 20 Hz) on lumbar and abdominal muscles could increase the muscle strength of the extensor muscle, whereas 40 Hz would decrease the extensor muscle endurance [8]. Moreover, the Iα inhibitory neurons in the drafting state could be activated by WBV (Less than or equal to 5 Hz), which could promote the reduction of single synaptic reflexes and relax tensed muscles [31]. High-intensity vibration would forcefully expose the muscle spindles to vibration stimulation, producing an inhibitory reflex, blocking muscle spindle transmitters, reducing the sensitivity of muscle spindle and causing muscle fatigue [32, 33]. The vibration frequency of lumbar abdomen is mostly below 20 Hz, and the results showed that such value could relieve pain in patients with CLBP and reduce the dysfunction index [16, 34]. In current studies, 15 Hz induces higher neuromuscular activity compared with 5 Hz and 10 Hz. Considering the overall evidence, 15 Hz vibration frequencies can lead to enhanced exercise benefits.

### Effect of different exercises on lumbar-abdominal muscle activation

Among the various exercises selected in this study, the optimum routines included plank for MF and ES, single bridge for AOE and V crunch for RA. Different muscles perform differently during exercises considering their own anatomical characteristics.

MF and ES are kinds of back muscles that maintain the spinal stability and balance. During plank exercise, the muscles are in static equal-length contraction, which would activate as many muscles, especially the deep stabilizing muscles such as the MF, as possible to maintain the stability of the spine [26]. MF contains rich proprioceptive receptors that enable the performance of delicate movements. When the spine suddenly becomes imbalanced, the MF would contract before the larger muscle groups adjust the displacement of individual vertebral segments. MF is a fundamental part to maintaining the normal biometric lines of the spine and enhancing lumbar stability [35, 36]. ES covers the lumbar and thoracic region; it is a large muscle group that maintains the stability of the spine. ES is a primary muscle for plank; both sides of ES contract to resist body gravity and keep the spine in a stable condition [36]. Previous studies showed that side stay and bridge exercises are good for back muscles, such as MF and ES, in WBV condition [26]. The most possible explanation for the conflicting results of this experiment could be the interaction effects between vibration frequencies and exercises on muscles. In general, WBV induced the greatest back muscle activation during plank training.

RA and AOE are both lumbar–abdominal muscles. During V crunch exercise, both ends of the body leave the ground, and all the gravity is concentrated on the abdominal muscles. These muscles require more muscle spindles to resist the load. On the other hand, the action of the V crunch is a centripetal contraction, whereas the RA is an active muscle requiring more strength. Atsushi et al. observed the effects of different platforms on trunk muscles during core stability training. The results showed that V crunch induced the highest RA activities on an unstable platform [37]. Vera-Garcia et al. observed that the degree of RA activation reached more than 10% of that of AOE under the V crunch exercise with unstable conditions [38]. Our findings were similar to those of previous studies. AOE is a large, thin and irregularly quadrilateral muscle located on the side of abdomen. During single bridge, one side of the upper limb and lower limb was off the ground; the AOE would then undertake more tasks to prevent the body from tilting. Furthermore, single bridge exercise induces more activities on AOE when compared with other exercises [39]. Therefore, in the case of WBV training, RA in the action of V crunch and AOE in the action of single bridge showed the highest value of RMS, implying that the V crunch and single bridge could better activate abdominal muscles.

### Clinical implications

Some clinical implications were provided in our study. First, WBV can effectively induce muscle activation in patients with CLBP, indicating that it could be an effective assisted intervention to improve muscle performance, such as muscle strength, proprioception and flexibility [40, 41]. Second, the different WBV parameters have different effects on the lumbar-abdominal sEMG. Exercise, frequency and their interactions should be considered during WBV training of CLBP patients. We determined the best combination of WBV intensity at 15 Hz and 2 mm and plank or single bridge or V crunch exercises. If you want to improve back muscle performance, Plank or single bridge should be the more preferred choice for these individuals. If you want to improve abdominal muscle performance, V crunch would be a good choice.

### Limitations

First, the results of this study could only be generalised to younger CLBP patients and could not represent all age groups with CLBP. Second, the selection of exercise featured certain limitations. Future research can expand the range of movements and observe the effect of different frequencies on the muscles in the case of dynamic movements. Third, amplitudes and frequencies are important factors for the effect of WBV training. Thus, future studies should include different amplitudes and additional frequencies.

## Conclusion

Adding WBV to exercise could increase muscle activation of lumbar–abdominal muscle in patients with CLBP. High vibration frequencies can lead to enhanced exercise benefits within an appropriate frequency range, and different exercises have diverse effects on various muscles. Our results revealed that 15 Hz induces higher neuromuscular activity than lower WBV frequencies (5 and 10 Hz). As for all lumbar–abdominal muscles, plank is suitable for the back muscles, single bridge for AOE and V crunch for RA.

## Data Availability

The datasets used and/or analysed during the current study are available from the corresponding author on reasonable request.

## Abbreviations

WBV: Whole body vibration
CLBP: Chronic low back pain
LBP: Low back pain
MF: Multifidus
ES: Erector Spinae
AOE: Abdominal Oblique Externus
RA: Rectus Abdominis
sEMG: Surface Electromyography
RMS: Root Mean Square
